# Will Small-Pox Protect from Vaccinia?

**Published:** 1886-01

**Authors:** 


### Will Small-Pox Protect from Vaccinia?

This question has been answered in the negative by the experience of a number of practitioners during the recent numerous vaccinations. Five patients who had well-marked pits of variola on their faces, have been vaccinated, on whom the virus operated, producing undoubted vaccinia. It is settled, then, that even if vaccinia will protect against or modify small-pox, the converse is not true.

---

We are happy to announce that the Buffalo Medical Library is an assured fact. As seen by a report from a member of the Board of Trustees, it is on a safe financial footing, and likely to be a great success in the near future. It is to be hoped that when the new Y. M. A. Library building is completed, that the Medical Library can find a permanent home there, and thus be accessible to the profession of the east and west sides.

